# Clinical-pathologic characteristics and response to neoadjuvant chemotherapy in triple-negative low Ki-67 proliferation (TNLP) breast cancers

**DOI:** 10.1038/s41523-022-00415-z

**Published:** 2022-04-20

**Authors:** Pooja Srivastava, Tiannan Wang, Beth Z. Clark, Jing Yu, Jeffrey L. Fine, Tatiana M. Villatoro, Gloria J. Carter, Adam M. Brufsky, Vikram C. Gorantla, Shannon L. Huggins-Puhalla, Leisha A. Emens, Thais Basili, Edaise M. da Silva, Jorge S. Reis-Filho, Rohit Bhargava

**Affiliations:** 1 Departments of Pathology, UPMC Magee-Womens Hospital, Pittsburgh, PA USA; 2 Departments of Medical Oncology, UPMC Magee-Womens Hospital, Pittsburgh, PA USA; 3 Department of Pathology, Memorial-Sloan Kettering Cancer Center, New York, NY USA; 4 Present Address: Keck School of Medicine of USC, Los Angeles, CA USA

**Keywords:** Breast cancer, Prognostic markers

## Abstract

Triple-negative breast cancers (TNBCs) often have a high Ki-67 proliferation index and respond favorably to neoadjuvant chemotherapy (NACT) with pathologic complete response (pCR) resulting in ~40% of cases. Nevertheless, morbidity/mortality remain high, mostly due to recurrence in patients with residual disease. In contrast, the incidence and clinical features of TNBC with low proliferation (TNLP), defined as TNBC with a Ki-67 index of ≤30% remains unknown. We report 70 cases of TNLP identified at our center from 2008 to 2018, including 18 treated with NACT. TNLP tumors represent <1% of all breast cancers, and ~5–10% of TNBCs. Ninety percent of carcinomas were grade I/II and 70% were either pure apocrine or showed apocrine differentiation. Fifty cases had available immunohistochemistry results; 80%, 84%, 22%, and 20% were positive for AR, INPP4B, nestin, and SOX10, respectively. With a median follow-up of 72 months, 14% experienced recurrence, and 11% died of breast cancer. The tumor stage was prognostic. Among 39 stage-I patients, 18 (46%) received chemotherapy, but this did not impact survival. There was a trend for improved recurrence-free survival with chemotherapy in stage-II patients. Of the 18 patients treated with NACT, 2 (11%) showed pCR; these were notable for either high stromal TILs or a high mitotic count despite a low Ki-67 index. TNLPs are enriched in low to intermediate-grade carcinomas with apocrine features. Due to overall good prognosis of stage-I TNLP and the lack of clear benefit of chemotherapy, de-escalation of chemotherapy may be considered in select patients with stage-I TNLP.

## Introduction

Breast cancer is the most common malignancy diagnosed in women^1–4^. Breast cancers which lack the expression of estrogen receptor (ER), progesterone receptor (PR), and amplification or overexpression of *ERBB2* (HER2) are classified as triple-negative breast cancer (TNBC). TNBCs represent ~15–20% of all breast cancers and are the most aggressive subtype with high tumor cell proliferation^2,5–10^. They are associated with a worse prognosis, with a high risk of local and distant recurrence and short overall survival^6,11–13^. TNBC, however, is an operational term and encompasses a rather heterogeneous group of tumors with at least four transcriptomic subtypes identified^5^.

When breast cancer molecular portraits were described in the 2000s, TNBCs were thought to be immunohistochemical (IHC) surrogates of basal-like breast cancers^14,15^. It has since become clear that, although there is significant overlap between TNBC and basal-like breast cancers, they are not synonymous^16,17^. Both triple-negative and basal-like breast cancers contain heterogeneous tumor morphologies with different prognoses. TNBCs not only contain the typical highly aggressive type of breast cancers but also many low-grade and less aggressive forms of breast cancers, such as adenoid cystic, secretory, mucoepidermoid, and low-grade adenosquamous^18,19^. In the last decade, TNBCs were initially molecularly sub-classified into basal-like 1 (BL1), basal-like 2 (BL2), mesenchymal (M), mesenchymal stem-like (MSL), immunomodulatory (IM), and luminal androgen receptor type (LAR)^20^. The BL1 and BL2 are typical high-grade TNBCs, with BL2 also showing additional growth factor signaling such as EGF pathway, NGF pathway, MET pathway, Wnt/beta-catenin, and IGF1R pathway. The IM subtype shows substantial overlap with the gene expression profile of “medullary” breast cancers. The M and MSL subtypes are enriched for metaplastic carcinomas (with low-proliferation seen in the MSL subtype) whereas LAR accounts for TNBCs with androgen receptor (AR) expression. This classification has more recently been refined from 6 to 4 (TNBCtype-4) tumor-specific subtypes (BL1, BL2, M, LAR)^21^. The second classification of Burstein et al. sub-classified TNBCs into four stable groups, basal-like immune activated (BLIA), basal-like immune-suppressed (BLIS), mesenchymal-like (MES), and luminal androgen receptor (LAR)^22^. There is significant overlap between Burstein and Lehmann classification with Lehmann classification identifying a basal-like group with growth factor signaling that may be resistant to chemotherapy and the Burstein group dividing the typical basal-like tumors into two groups based on the amount of tumor-infiltrating lymphocytes. Both classifications, however, identify an LAR subtype that is seemingly less aggressive but with a reported lower rate of pathologic complete (pCR) response to neoadjuvant chemotherapy (NACT). This is not an entirely novel finding, as AR expression in ER-negative tumors (regardless of HER2 status) has been specifically associated with tumors showing apocrine differentiation^23–25^. Apocrine cancers can be of low or high grade, and prognosis may vary in this LAR subgroup depending on grade and stage.

A relatively consistent feature of most TNBCs is high tumor cell proliferation as measured by the Ki-67 antibody^26^. More than three-quarters of TNBCs show a Ki-67 proliferation index of >50%. The correlation between a high Ki-67 proliferation index and TNBC is so strong that a TNBC with a low proliferation index is viewed with suspicion by clinicians. Although some special histologic subtypes of TNBC (adenoid cystic, secretory, and low-grade adenosquamous) and the molecular LAR subtype are known to have low proliferation, the morphologic spectrum of TNBC with low proliferation (TNLP) has not been systematically evaluated. Thus, optimal treatment and prognosis for TNLP poses a significant challenge for medical oncologists. Here, we describe the clinicopathologic features and clinical outcomes of TNLPs at our center to provide insight in managing patients with such tumors.

## Results

We identified 70 cases of primary TNLP over 11 years (2008–2018). Based on the case volume at our institution, we estimate that TNLP tumors represent <1% of all breast cancers and ~5–10% of TNBCs. The patient and tumor characteristics are summarized in Table 1. The median age at diagnosis was 66 years, and the median tumor size was 1.6 cm. Regional lymph node involvement was identified in 24% of the cases. Ninety percent of the tumors were grade I or II. The most common histopathological subtype was apocrine tumors with 70% cases (49 of 70 cases) being either pure apocrine carcinomas or carcinomas with apocrine differentiation.

**Table 1:** Patient and tumor characteristics (*n* = 70).

Age in years	
Mean	66 years
Median	66 years
Range	36–98 years
Procedure	
Segmental	42 (60%)
Total mastectomy	23 (33%)
Modified radical mastectomy	5 (7%)
Tumor size in cm	
Mean	2.2 cm
Median	1.6 cm
Range	0.2–10.5 cm
Tumor grade	
I	10 (14%)
II	53 (76%)
III	7 (10%)
Nottingham score	
4	1 (2%)
5	9 (13%)
6	27 (38%)
7	26 (37%)
8	5 (7%)
9	2 (3%)
LN status	
Negative	51 (73%)
Positive	17 (24%)
Not available	2 (3%)
pT stage	
1	44 (63%)
2	18 (26%)
3	8 (11%)
pN stage	
0	51 (73%)
1	11 (16%)
2	4 (6%)
3	2 (2.5%)
Unknown	2 (2.5%)
AJCC Stage^a^	
I	39 (56%)
II	23 (33%)
III	7 (10%)
IV	1 (1%)
HER2 immunohistochemistry	
Score 0	13 (19%)
Score 1+	20 (28%)
Score 2+/in-situ hybridization negative	37 (53%)
Ki-67 index	
1–10%	33 (47%)
11–20%	21 (30%)
21–30%	16 (23%)
Stromal TILs	
1–10%	49 (70%)
11–30%	14 (20%)
31% or more	7 (10%)
Tumor type	
Apocrine	49 (70%)
Histiocytoid	5 (7%)
No special type	7 (10%)
Other	9 (13%)
Neoadjuvant chemotherapy	
No	52 (74%)
Yes	18 (26%)
Response to neoadjuvant chemotherapy (*n* = 18)	
pCR	2 (11%)
Residual Cancer Burden 1	0 (0%)
Residual Cancer Burden 2	10 (56%)
Residual Cancer Burden 3	6 (33%)
Radiation	
No	20 (29%)
Yes	47 (67%)
Unknown	3 (4%)
Systemic chemotherapy	
No	28 (40%)
Yes	42 (60%)
Recurrence	
No	60 (86%)
Yes	9 (12%)
Metastasis at diagnosis	1 (2%)
Recurrence type	
No recurrence	60 (86%)
Loco-regional	2 (2.5%)
Distant only	5 (7%)
Local+Distant	2 (2.5%)
Metastasis at diagnosis	1 (2%)
Vital status	
Alive	57 (82%)
Died of other causes	5 (7%)
Died of breast cancer	8 (11%)

^a^Two cases with unknown pN stage were considered as node negative for AJCC staging due to negative clinical nodal status.

**Fig. 1:**
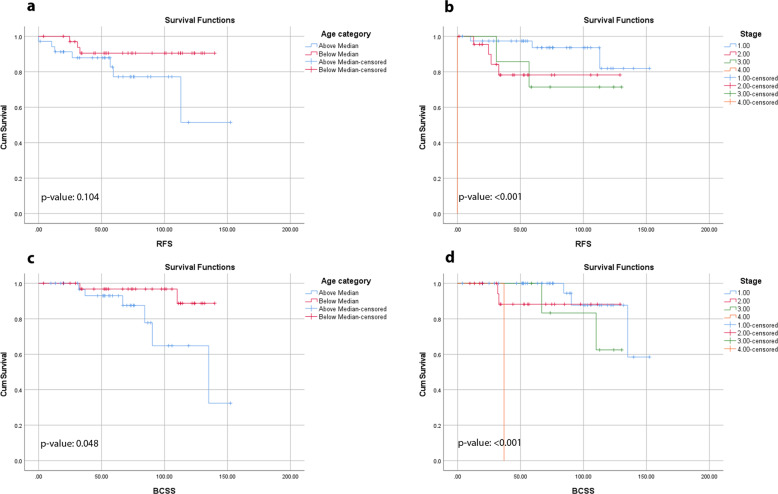
Survival based on age and AJCC stage. Kaplan–Meier survival curves for recurrence-free (RFS) and breast cancer-specific survival (BCSS) for age and AJCC stage (**a** RFS for age, log-rank test *p*-value 0.104; **b** RFS for stage, log-rank test *p*-value < 0.001; **c** BCSS for age, log-rank test *p*-value 0.048; **d** BCSS for stage, log-rank test *p*-value < 0.001).

With an average follow-up of 73 months (median 72 months), the RFS was 86% and the BCSS of 89%. Survival for each variable was assessed by KM survival analysis. The log-rank test *p*-values for each variable are shown in Table 2. Age and stage RFS and BCSS curves are shown in Fig. 1. Longer RFS was associated with limited surgical procedure (segmental/total better than modified radical mastectomy), negative lymph node status (pN0), and AJCC stage I (supplementary Fig. 1). Longer BCSS was associated with younger age (age less than median age in this study, i.e., <66 years), limited surgical procedure (segmental/total better than modified radical mastectomy), negative lymph node status (pN0), and AJCC stage I (supplementary Fig. 2). The tumor clinical-pathologic features on patients who recurred are summarized in Table 3.

**Table 2:** Survival assessed by Kaplan–Meier survival analysis using log-rank test for each variable.

Variables	Data	RFS (log-rank test *p*-value)	BCSS (log-rank test *p*-value)
Age in years		0.104	0.048^a^
Above median age of 66 years	35 (50%)		
Below median age of 66 years	35 (50%)		
Range	36–98 years		
Procedure		0.013^a^	0.024^a^
Segmental	42 (60%)		
Total mastectomy	23 (33%)		
Modified radical mastectomy	5 (7%)		
Tumor grade		0.521	0.133
I	10 (14%)		
II	53 (76%)		
III	7 (10%)		
Nottingham score		0.727	0.591
4–6	37 (53%)		
7–9	33 (47%)		
LN status		0.028^a^	0.036^a^
Negative	51 (73%)		
Positive	17 (24%)		
Not available	2 (3%)		
pT stage		0.081	0.061
1	44 (63%)		
2	18 (26%)		
3	8 (11%)		
pN stage		0.001^a^	0.005^a^
0	51 (73%)		
1	11 (16%)		
2	4 (6%)		
3	2 (2.5%)		
Unknown	2 (2.5%)		
AJCC Stage		0.000^a^	0.000^a^
I	39 (56%)		
II	23 (33%)		
III	7 (10%)		
IV	1 (1%)		
HER2 immunohistochemistry		0.651	0.860
Score 0	13 (19%)		
Score 1+	20 (28%)		
Score 2+/FISH-negative	37 (53%)		
Ki-67 index		0.942	0.508
1–10%	33 (47%)		
11–20%	21 (30%)		
21–30%	16 (23%)		
Stromal TILs		0.449	0.492
1–10%	49 (70%)		
11–30%	14 (20%)		
31% or more	7 (10%)		
Tumor type		0.977	0.576
Apocrine	49 (70%)		
Histiocytoid	5 (7%)		
No special type	7 (10%)		
Other	9 (13%)		
Neoadjuvant chemotherapy		0.265	0.256
No	52 (74%)		
Yes	18 (26%)		
Response to neoadjuvant chemotherapy (*n* = 18)		0.199	0.60
Residual Cancer Burden 0	2 (11%)		
Residual Cancer Burden 1	0 (0%)		
Residual Cancer Burden 2	10 (56%)		
Residual Cancer Burden 3	6 (33%)		
Radiation		0.534	0.869
No	20 (29%)		
Yes	47 (67%)		
Unknown	3 (4%)		
Systemic chemotherapy		0.920	0.940
No	28 (40%)		
Yes	42 (60%)		

*RFS* recurrence-free survival, *BCSS* breast cancer-specific survival, *FISH* fluorescence in-situ hybridization.

^a^Statistically significant.

**Table 3:** Clinical features of cases that recurred.

Case deid#	Age in years	Type of recurrence	Time to rec (months)	Time to distant rec (months)	Vital status	Site(s) of distant rec	Stage at dx	Nott score
Rec1	69	LR+Distant	113	131	Died of BC	LNs, Brain	I	6
Rec2	78	Distant	59	59	Died of BC	Bone, lung	I	4
Rec3	54	Distant	25	25	Died of BC	Bone	II	6
Rec4	61	Distant	31	31	Died of BC	Pleura	III	7
Rec5	73	LR+Distant	10	53	Died of BC	LNs, lung, liver	I	7
Rec6	67	Mets at dx	0	0	Died of BC	Lung, spine	IV	8
Rec7	68	Distant	57	57	Died of BC	Bone, pleura	III	6
Rec8	56	LR	33	NA	Alive, no cancer	None	II	7
Rec9	97	Distant	27	27	Died of BC	Lung	II	8
Rec10	81	LR	13	NA	Died of OC	None	II	6

*Deid* de-identified, *LR* loco-regional, *BC* breast cancer, *OC* other causes, *LN* lymph node, Rec recurrence, *Dx* diagnosis, *Nott* Nottingham, *NA* not applicable.

**Fig. 2:**
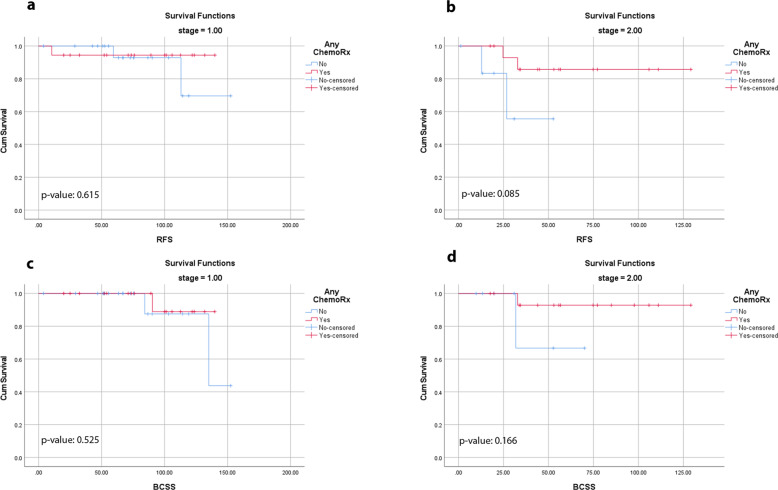
Survival based on chemotherapy use in stage I and II patients. Recurrence-free (RFS) and breast cancer-specific survival (BCSS) in stage-I and II patients based on whether they received chemotherapy or not (**a** RFS in stage-I patients for chemotherapy, log-rank test *p*-value 0.615; **b** RFS in stage-II patients for chemotherapy, log-rank test *p*-value 0.085; **c** BCSS in stage-I patients for chemotherapy, log-rank test *p*-value 0.525; **d** BCSS in stage-II patients for chemotherapy, log-rank test *p*-value 0.166).

We also assessed the effect of chemotherapy on survival based on nodal status and AJCC stage. Overall, 60% of the patients received chemotherapy with increasing chemotherapy use in higher-stage patients. There was no difference in RFS (log-rank test *p*-value of 0.914 for lymph node negative and 0.541 for lymph node positive) or BCSS (log-rank test *p*-value of 0.435 for lymph node negative and 0.523 for lymph node positive) whether a patient received chemotherapy or not based on nodal status. Chemotherapy was administered to 18 of 39 stage-I patients (46%), 16 of 23 stage-II patients (70%), and all stage III and IV patients (seven stage-III and one stage-IV). Given that some early-stage patients received chemotherapy and some did not, we attempted to identify the benefit of chemotherapy in stage-I and II patients. There was no difference in RFS or BCSS whether a patient received chemotherapy or not in patients with stage-I disease (Fig. 2). There was a numerical trend for improved RFS in stage-II patients who received chemotherapy (Fig. 2). Chemotherapy administration was found not to alter significantly the BCSS in patients with stage-II disease (Fig. 2).

**Fig. 3:**
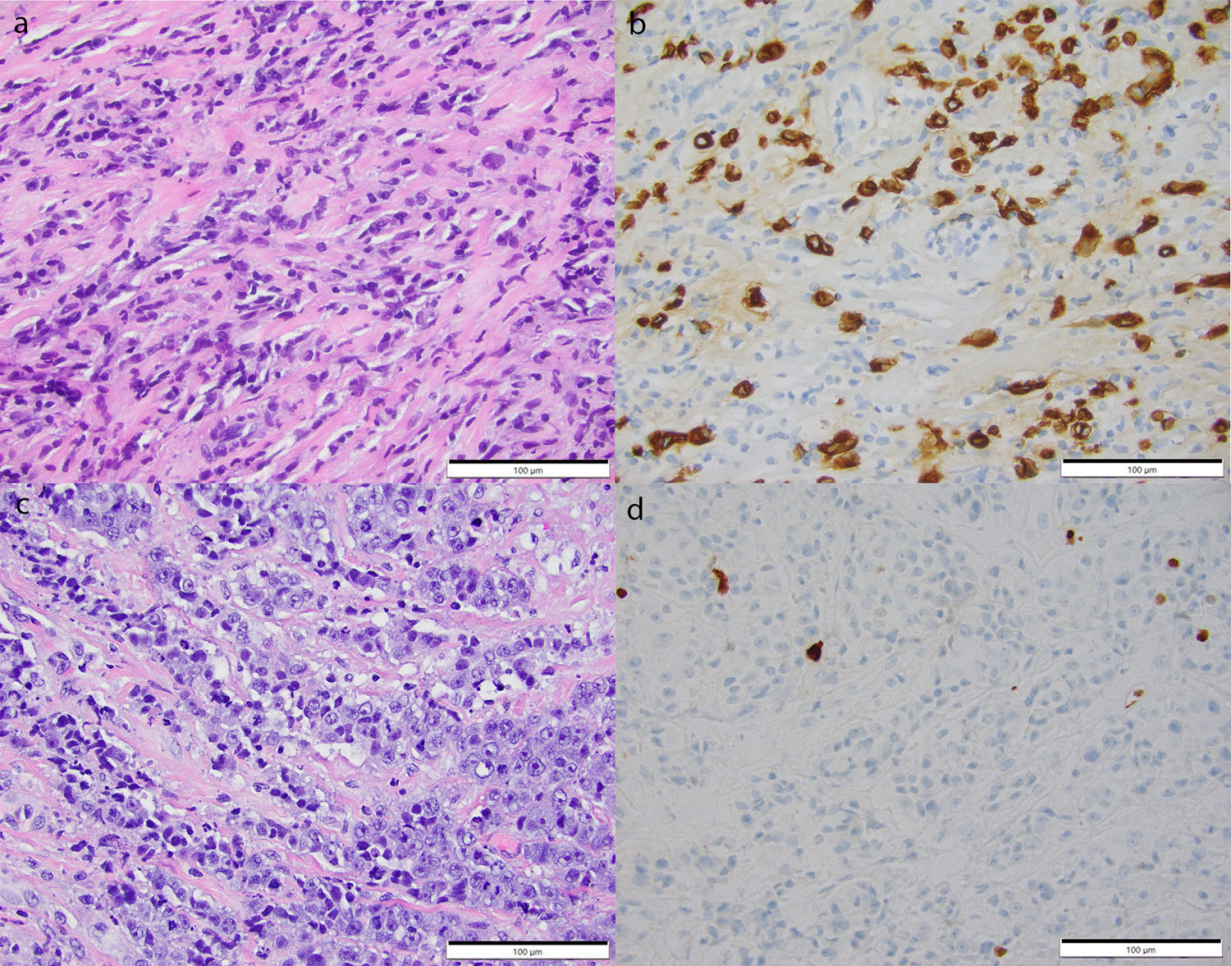
Cases from two patients who responded completely to neoadjuvant chemotherapy. First case with high stromal tumor-infiltrating lymphocytes as seen on pre-therapy core biopsy (**a** H&E, **b** Cytokeratin AE1/AE3 highlighting the cancer cells with background unstained lymphoid cells). Second case with low Ki-67 proliferation index but high mitotic activity (**c** H&E, **d** Ki-67 immunohistochemical stain). Scale bar = 100 µ.

Eighteen of the 70 patients were treated with NACT. Of these 18 patients, two (11%) evolved to pCR. Both were clinical stage-II tumors. One of these cases showed high stromal tumor-infiltrating lymphocytes (50%) and the other case showed a very high mitotic activity despite having a low Ki-67 proliferation index (Fig. 3). Both patients did not experience recurrence and were alive at the last follow-up (106 and 129 months).

Multivariable Cox regression analysis could not be performed for RFS since only nodal status, pN stage, AJCC stage, and final surgical procedure were statistically significant by log-rank test on univariable analysis and all these variables are known to show multicollinearity. To avoid multicollinearity issues for BCSS, age was included with either nodal status or with the stage (2-tier) for multivariable Cox regression analysis. Both younger age (i.e., age less than 66 years; *p*-value: 0.031, hazard ratio: 0.061, 95% CI: 0.005–0.776) and negative nodal status (*p*-value: 0.018, hazard ratio: 0.057, 95% CI: 0.005–0.608) in the first model and younger age (*p*-value: 0.010, hazard ratio: 0.038, 95% CI: 0.003–0.459) and stage I (*p*-value: 0.011, hazard ratio: 0.046, 95% CI: 0.004–0.491) in the second model were found to be statistically significantly associated with longer BCSS.

**Fig. 4:**
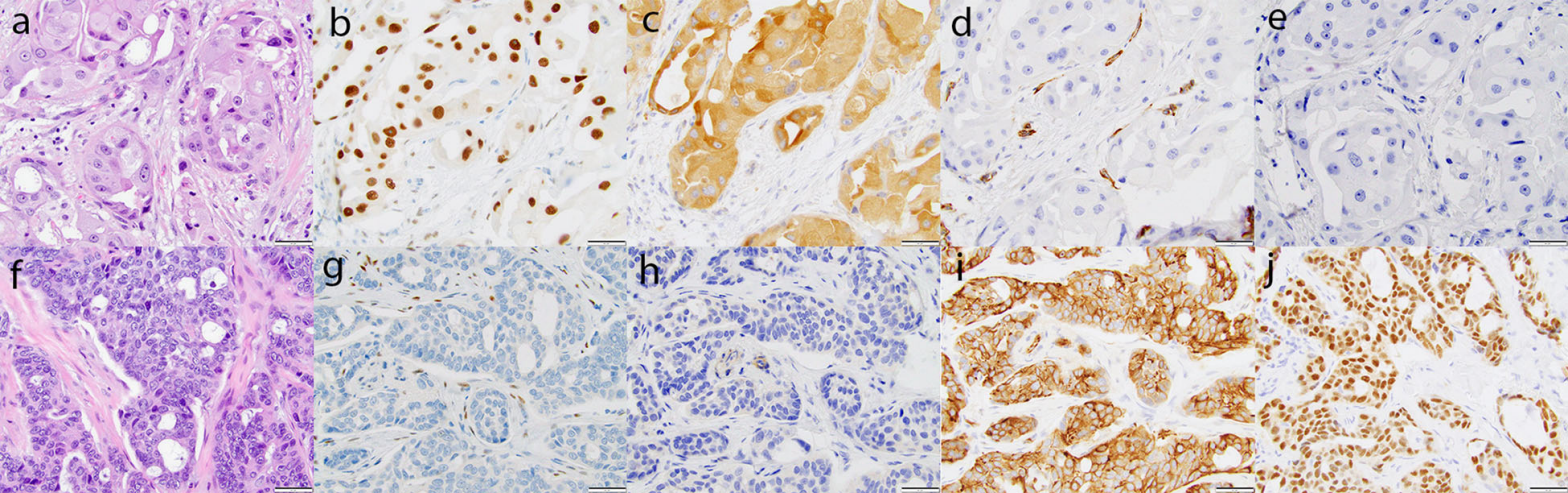
Immunohistochemical expression of luminal and basal markers. An apocrine carcinoma (**a**) showing diffuse-strong reactivity for AR (**b**) and INPP4B (**c**) but is negative for nestin (**d**) and SOX10 (**e**) while this no special type TNBC (**f**) is negative for AR (**g**) and INPP4B (**h**) but positive for nestin (**i**) and SOX10 (**j**). Scale bar = 50 µ.

The predominant subtype seen in TNLP was either pure apocrine carcinoma or carcinoma with apocrine features (70%). The patient and tumor features of apocrine carcinomas and carcinomas with apocrine features are provided in supplementary table 1. The tumor histology was correlated with the IHC profile (Table 4). E-cadherin/p120 dual stain was performed on all 70 cases. Most apocrine tumors showed ductal profiles with E-cadherin and p120 (44 of 49 cases, 90%), whereas most histiocytoid tumors showed lobular profiles (4 of 5 cases, 80%). Of the 50 cases (represented on tissue microarray) with IHC results, AR was positive in 40 (80%), INPP4B in 42 (84%), nestin in 10 (20%), and SOX10 in 11 (22%). AR reactivity was seen mainly in pure apocrine carcinomas or carcinomas with apocrine or histiocytoid features (Table 4). The AR-positive cases often showed diffuse strong immunoreactivity (median and range of H-scores on positive cases: 285, 70–300). INPP4B staining correlated with AR expression; however, the H-scores were slightly lower (median and range of H-scores on positive cases: 130, 10–220). GCDFP-15 reactivity mirrored AR and INPP4B expression. GCDFP-15 was positive in 40 of 50 cases (80%). All apocrine carcinomas (26/26), histiocytoid carcinomas (5/5), carcinomas with apocrine differentiation (8/8), and one of 6 (1/6) carcinoma of no special type were positive for GCDFP-15. The reactivity for GCDFP-15 was diffuse and strong (median and range of H-scores on positive cases: 245; 50–300). An inverse correlation was identified between luminal (AR and INPP4B) and basal (nestin and SOX10) markers (Fig. 4). Nestin was positive in ten cases and SOX10 in 11 cases. The H-scores for nestin on positive cases ranged from 5–240 with a median H-score of 33. The H-scores for SOX10 on positive cases ranged from 120–280, with a median H-score of 230. No significant differences were noted for RFS or BCSS based on any IHC result.

**Table 4:** Expression of luminal and basal markers in triple negative low proliferation (TNLP) tumors.

Morphology	NA for staining (not on TMA)	Available for staining (TMA)	AR positive	INPP4B positive	Nestin positive	SOX10 positive
Adenoid cystic (*n* = 3)	2	1	0	0	1	1
Atypical MGA-like (*n* = 1)	0	1	1	1	1	1
CA-apocrine features (*n* = 14)	7	7	7	7	0	0
Apocrine (IDC) (*n* = 30)	7	23	22	23	0	1
Apocrine (ILC) (*n* = 3)	0	3	3	3	0	0
Apocrine (Mixed) (*n* = 2)	1	1	1	1	0	0
Histiocytoid (IDC) (*n* = 1)	0	1	1	1	0	0
Histiocytoid (ILC) (*n* = 4)	0	4	4	1	0	0
LG adenosquamous (*n* = 3)	0	3	0	3	3	3
Micropapillary mucinous (*n* = 1)	1	0	NA	NA	NA	NA
No special type (*n* = 7)	1	6	1	2	5	5
Squamous (*n* = 1)	1	0	NA	NA	NA	NA
Total *(n* = 70)	20	50	40	42	10	11

*NA* not available, *MGA* microglandular adenosis, *TMA* tissue microarray, *AR* androgen receptor, *CA* carcinoma, *IDC* invasive ductal carcinoma, *ILC* invasive lobular carcinoma, *LG* low grade.

## Discussion

Due to lack of ER/PR and HER2 overexpression, early TNBCs lack a specific target, and therefore, all patients diagnosed with TNBC, stage IB and higher are offered chemotherapy. With few exceptions, little consideration is given to tumor histology, grade, or Ki-67 proliferation index. Pathologists who routinely perform Ki-67 IHC are aware that most TNBCs show a very high proliferation index (often > 50%). When a TNBC shows a low proliferation index, it automatically triggers a review of morphology and other receptor results. In most cases, the review is quite informative and confirms the overall low/intermediate grade of the tumor and often a histologic subtype different from a usual TNBC. Nevertheless, a systematic review of TNBC with low proliferation has not previously been reported. Our study confirms that TNLP tumors are mostly low grade (1 or 2) and frequently show unique tumor histologies distinct from no special type. The most frequent histology seen is pure apocrine carcinoma or carcinomas with apocrine differentiation. Other histologic subtype/morphologies represented in our case cohort were histiocytoid, low-grade adenosquamous, adenoid cystic, and carcinoma showing an atypical microglandular adenosis-like pattern. A minority of the tumors were no special type carcinomas, but only one of the 7 no special type carcinomas were grade III. This patient received NACT and achieved pCR (see below). Our exploratory immunohistochemical analysis demonstrates that most apocrine carcinomas show membranous E-cadherin and p120 reactivity (hence “ductal”), whereas most histiocytoid carcinomas demonstrate a lack of E-cadherin staining with cytoplasmic p120 expression (hence “lobular”). We also confirm that luminal marker expression (AR and INPP4B) is associated with tumors showing apocrine differentiation and that there is a strong inverse correlation between luminal marker reactivity (AR, INPP4B) and basal marker reactivity (nestin, SOX10). Although no survival differences were noted for IHC markers, it is to be noted that all tumors in this study were of TNLP type. This staining panel should be further explored in subtyping of TNBC where along with morphology and Ki-67, it may be of use in determining prognostically useful categories within TNBCs.

Diagnosis of TNBC on core needle biopsy often triggers a referral to a medical oncologist for consideration of NACT. Although not all TNBCs are the same, a diagnosis of TNLP poses a dilemma for the oncologist, especially when the clinical tumor stage is I or II. A pathologist’s input regarding special tumor morphology may inform the decision, but pathologists are generally not involved in this decision-making process. Although only two of 18 patients achieved pCR to NACT, our study provides some insight on the use of NACT in TNLPs. One of two cases with pCR showed high stromal TILs (50%). It is noteworthy that this high level of stromal TILs in a TNLP is uncommon since >30% of stromal TILs were seen only in 10% of the TNLPs in this study (Table 1). It is, therefore, a good practice to report stromal TILs on core needle biopsy samples with TNBC diagnosis regardless of the Ki-67 proliferation index. The second case with pCR showed high mitotic activity despite a low Ki-67 proliferation index. Generally, mitotic activity and Ki-67 proliferation index show excellent linear correlation, but discrepancies do occur. This finding implies that both morphology and ancillary testing should be taken into consideration for making therapy decisions. We want to further emphasize that these findings stemmed from a hypothesis generating, exploratory analysis and that further validation in larger, independent cohorts would be required.

Although TNLP is morphologically heterogeneous, it can be a useful clinical category for proper patient management. For the pathologist, this should trigger a reappraisal of the tumor morphology to explain the low proliferation in a TNBC. For the surgeon or medical oncologist, caution is advised for using neoadjuvant chemotherapy. Consultation with a breast pathologist or presentation at an interdisciplinary tumor board may be useful in making a final management recommendation. Our data suggest that chemotherapy may not be highly beneficial in stage-I TNLP tumors. Oncologists may not be able to withhold chemotherapy in higher-stage patients; however, for clinical stage I or patients with an unclear stage, it may be better to proceed with primary surgery in TNLP cases and then decide about chemotherapy when all information is available after primary resection.

A study similar to ours has not been previously reported. However, there are several studies regarding the Ki-67 proliferation rate in TNBC and its correlation to clinical outcome. Kubouchi et al. studied 51 cases of stage-I/II TNBC and suggested that apocrine type TNBC with low Ki-67 proliferation index may not benefit from neoadjuvant chemotherapy^4^. A meta-analysis of 35 studies and 7716 enrolled patients showed that a high Ki-67 proliferation index was significantly associated with poor disease-free and overall survival in resected TNBC. With a cut-off value of ≥40% Ki-67 proliferation index, the pooled hazard ratio was 2.30 (95% CI 1.54–3.44, *p* < 0.001) for disease-free survival and 2.95 (95% CI 1.67–5.19, *p* < 0.001) for overall survival^27^. Zhu et al. studied 1800 cases of early-stage TNBC to identify the best cut-off value for the Ki-67 proliferation index with regards to prognosis (disease-free survival and overall survival)^28^. They identified the most relevant cut-off value to be 30%. For our study, we used a cut-off of 30% Ki-67 proliferation index. Our initial expectation was to identify a large number of salivary gland-like tumors in this cohort, but the actual numbers show enrichment for the low/intermediate-grade apocrine type tumors. Although grade 3 apocrine tumors do exist, those were likely excluded from this group of TNLP. AR + TNBC has been regarded as IHC correlate of the LAR molecular subtype. The reported data is still immature regarding LAR subtype and response to neoadjuvant chemotherapy and overall prognosis. In the revised Lehmann TNBCtype-4 classification^21^, the pathologic complete response rate after neoadjuvant chemotherapy was 29% for the LAR subtype compared to 41% for BL1 and 18% for BL2. No statistically significant difference in overall survival was identified, however, based on the molecular classes. Other authors have reported that apocrine type of TNBCs show low histological grade and Ki-67 proliferation index and most likely corresponds to the “LAR” subtype on molecular characterization^4,21,22,29–32^. Our findings support the contention that morphology (histology and grade) and immunohistochemistry (Ki-67 and AR) may identify the subgroup within the “LAR” subtype that are unlikely to derive benefit from NACT and possibly adjuvant chemotherapy. Further, when combined with the anatomic stage, these histologic and IHC parameters can also help in identifying TNBC patients with an excellent prognosis.

Androgen receptor is known to be expressed in up to 90% of breast cancers and around 30% of TNBCs. In our study, AR reactivity was seen mainly in pure apocrine carcinomas or carcinomas with apocrine or histiocytoid features which is compatible with the findings reported by other investigators that molecular apocrine breast cancer is negative for ER and usually expresses AR and FOXA1^4,33,34^. However, 10% of these tumors lack FOXA1 expression^20^. Some authors have also reported that chemosensitive TNBC tends to show a lower expression of AR and FOXA1^6^. Whilst there is evidence that AR-positive TNBCs are usually less aggressive and have a better clinical outcome^35–42^, other studies have provided data contradicting this hypothesis^20,34,43,44^. Recognizing the subtypes of TNBC is, however, of importance for therapy decision-making^4,45,46^; we contend that prospective studies to test whether early-stage TNLPs should be treated differently than other TNBCs are warranted. Anti-androgen therapy can be particularly beneficial in early-stage AR + TNBCs. As these are low-risk patients with favorable prognosis, one can avoid overtreatment. AR + TNBCs frequently harbor *PIK3CA* mutations and emerging evidence suggests that a combination of an AR antagonist and PI3K inhibitor may result in higher clinical benefit than anti-AR therapy alone in AR + TNBC^47,48^. This should be an area of further study. Another potential therapeutic avenue that can be explored in these tumors is anti-HER2 antibody drug conjugates such as trastuzumab-deruxtecan, which has shown promising preliminary antitumor activity in patients with HER2-low tumors^49^. Over 80% of the tumors in our study cohort would qualify as HER2-low (28% cases with IHC score 1+ and 53% cases IHC score 2+ with lack of amplification, see Table 1).

One limitation of our study is that the TNLP cohort largely represented apocrine carcinomas/carcinomas with apocrine differentiation and other special subtype tumors were not well represented. We did not intend to select one morphological subtype over others in this consecutive series, but apocrine tumors happen to be the most frequent. Due to the nature of the criteria used, it is obvious that many high-grade apocrine carcinomas were likely excluded. This may also explain the rather good prognosis of apocrine carcinomas in this study. Further validation in larger, independent cohorts, including both apocrine TNLPs and high-grade/highly-proliferative apocrine carcinomas is required to confirm our findings.

In summary, we describe the clinical-pathologic features of a unique dataset of TNBCs with low proliferation. These TNLP tumors are enriched in low to intermediate-grade apocrine tumors, which demonstrate a “luminal-like” profile by IHC and are negative for “basal” markers. In contrast to usual forms of TNBC, patients with TNLP tumors are slightly older, have smaller tumor sizes at diagnosis, and lower tumor grades. Due to the overall good prognosis of stage-I patients and lack of clear benefit of chemotherapy in early-stage disease, de-escalation of chemotherapy may be considered in select stage-I triple-negative tumors with low Ki-67 proliferation index.

## Methods

### Case selection

After obtaining institutional review board approval, we queried our institutional pathology database from 2008 to 2018 and identified primary consecutive TNBC cases with a Ki-67 proliferation index of ≤30% (TNBC with lower proliferation index or TNLP). All locally recurrent cases were excluded. Ki-67 is routinely performed on all primary invasive breast carcinomas at our institution since 2008. Estrogen receptor (clone SP1), progesterone receptor (clone 1E2), HER2 (clone 4B5), and Ki-67 (clone 30–9) assays were performed and reported at the time of diagnosis. ER, PR, and HER2 results were reported according to the American Society of Clinical Oncology/College of American Pathologist (ASCO/CAP) guidelines. *HER2* fluorescence in-situ hybridization assay was performed on HER2 IHC 2+ cases to confirm HER2 negative status. Ki-67 was repeated on resection specimens when available to confirm the results of testing on core biopsies. Cases with a Ki-67 proliferation index of >30% were excluded. These criteria yielded 70 cases of TNLP, of which 18 cases were treated with NACT. Slides were reviewed on all cases for histologic classification. We retrospectively reviewed patient charts and recorded the clinical features of these 70 cases. Recurrence-free and breast cancer-specific survival (RFS and BCSS) analysis was performed for each of the clinical-pathologic variables. The response to NACT was also analyzed.

### Immunohistochemistry

All 70 cases were analyzed immunohistochemically with dual E-cadherin/p120 stain. Fifty cases had sufficient tumor for tissue microarray construction and immunohistochemical (IHC) analysis with androgen receptor (AR), INPP4B, nestin, and SOX10. The four marker panel (AR, INPP4B, nestin, SOX10) was utilized to identify whether TNLP tumors show a luminal-like profile or basal-like profile. Studies have shown that the expression of nestin and loss of INPP4B is a robust marker of basal-like breast cancer^50,51^. AR is an established marker of molecular LAR type and SOX10 has been shown to stain up to 70% of TNBCs of the usual type^21,52^. Therefore, the reactivity for AR and INPP4B was considered a luminal-like profile, while reactivity for nestin and SOX10 was considered a basal-like profile. Additionally, gross cystic disease fluid protein-15 (GCDFP-15) staining was also performed to determine its correlation with apocrine morphology and AR staining. The antibodies and the protocol used in this study are as follows: E-cadherin (Clone: 36; Vendor: Ventana, Tucson, AZ; Dilution: ready to use [RTU], Pre-treatment: CC1-S, Detection: Ultraview; Staining platform: Ventana Benchmark Ultra), p120 (Clone: 98; Vendor: BD Biosciences, Franklin Lakes, NJ; Dilution: 1:200; Pre-treatment: CC1-S; Detection: Ultraview; Staining platform: Ventana Benchmark Ultra), AR (Clone: SP107; Vendor: Ventana, Tucson, AZ; Dilution: RTU; Pre-treatment: CC1-M; Detection: Optiview; Staining platform: Ventana Benchmark Ultra), INPP4B (Clone: D9K1B; Vendor: Cell Signaling Technology, Danvers, MA; Dilution: 1:100; Pre-treatment: ER2, 40'; Detection: DAB Refine; Staining platform: Leica BOND III); Nestin (Clone: 10C2; Vendor: Cell Marque, Danvers, MA; Dilution: 1:50; Pre-treatment: ER2, 20'; Detection: DAB Refine; Staining platform: Leica BOND III); SOX10 (Clone: BC34; Vendor: Biocare Medical; Dilution: RTU; Pre-treatment: ER2, 20'; Detection: DAB Refine; Staining platform: Leica BOND III); GCDFP-15 (Clone:23A3; Vendor: Leica Biosystems; Dilution: RTU; Pre-treatment: ER1, 20'; Detection: Bond Polymer Refine Detection; Staining platform: Leica BOND III). AR and SOX10 localize to the nucleus, whereas INPP4B and nestin display cytoplasmic expression. GCDFP-15 shows cytoplasmic expression. An H-score of 1 or higher was considered a positive result for all IHC markers.

### Statistical analysis

RFS and BCSS were analyzed via Kaplan–Meier (KM) survival analysis for multiple independent variables as noted in Table 2 (age, final surgical procedure, tumor grade, Nottingham score, nodal status, pT stage, pN stage, AJCC stage, HER2 IHC score, Ki-67 proliferation index, histologic types, administration of neoadjuvant chemotherapy, response to NACT, administration of radiation, administration of systemic chemotherapy). Kaplan–Meier (KM) survival analysis for RFS and BCSS was also performed on 50 cases for various IHC markers (AR, INPP4B, Nestin, and SOX10). A log-rank test was used to compare KM curves. A *p*-value < 0.05 was considered significant. The variables showing statistically significant differences in survival by the log-rank test were included for multivariable Cox proportion hazard regression analysis. Statistical analysis was performed using IBM SPSS Statistics for Windows, Armonk, NY: IBM Corp.

### Reporting Summary

Further information on research design is available in the Nature Research Reporting Summary linked to this article.

## Supplementary information

Reporting summary

Supplementary figures and table

## Data Availability

The H&E and immunohistochemistry datasets generated and analyzed during the current study are not publicly available but can be made available upon reasonable request, following ethics committee approval and a data transfer agreement, to guarantee the General Data Protection Regulation.
