# Supplementary material for: Clinical-pathologic characteristics and response to neoadjuvant chemotherapy in triple-negative low Ki-67 proliferation (TNLP) breast cancers
Source: NPJ Breast Cancer. 2022 Apr 20;8:51. doi: 10.1038/s41523-022-00415-z (PMC9021249; doi:10.1038/s41523-022-00415-z)
Supplement: Supplementary file 2 — Supplementary figures and table [file 41523_2022_415_MOESM2_ESM.pdf]

Supplementary figure 1:

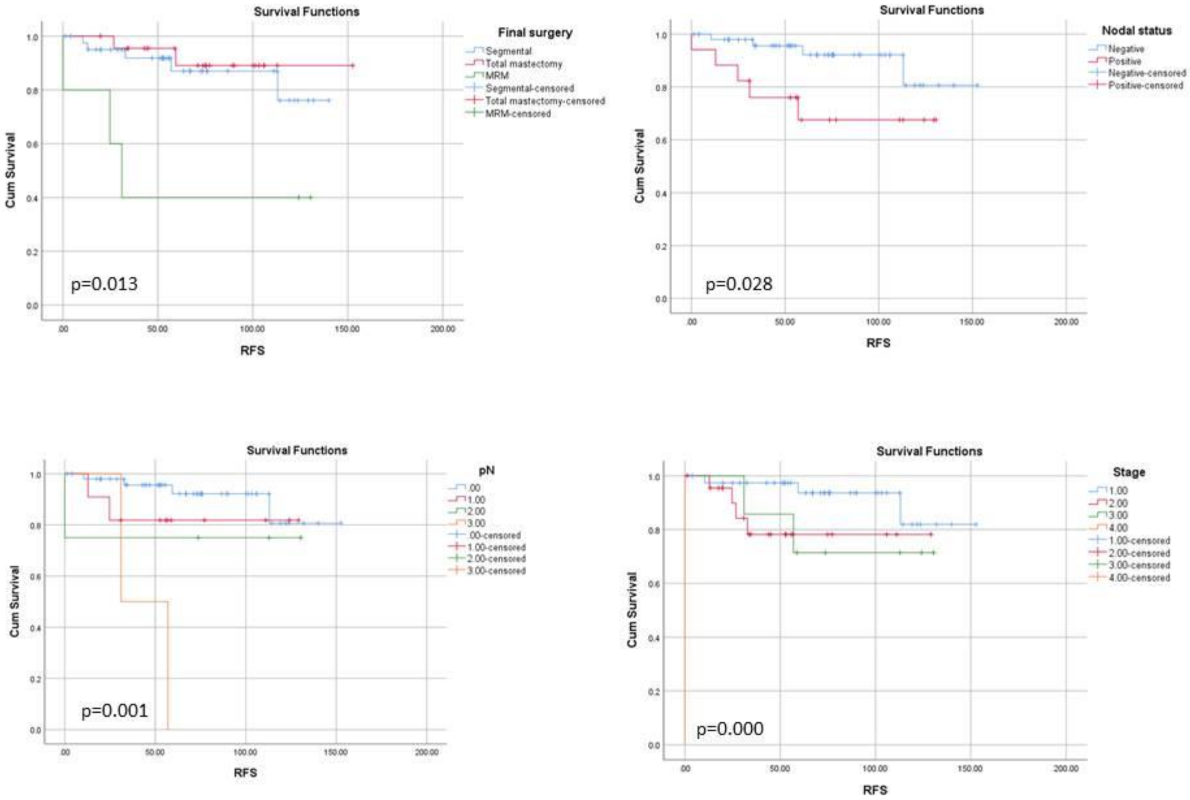


Supplementary figure 1: Variables significantly associated with recurrence-free survival (RFS)

Supplementary figure 2:

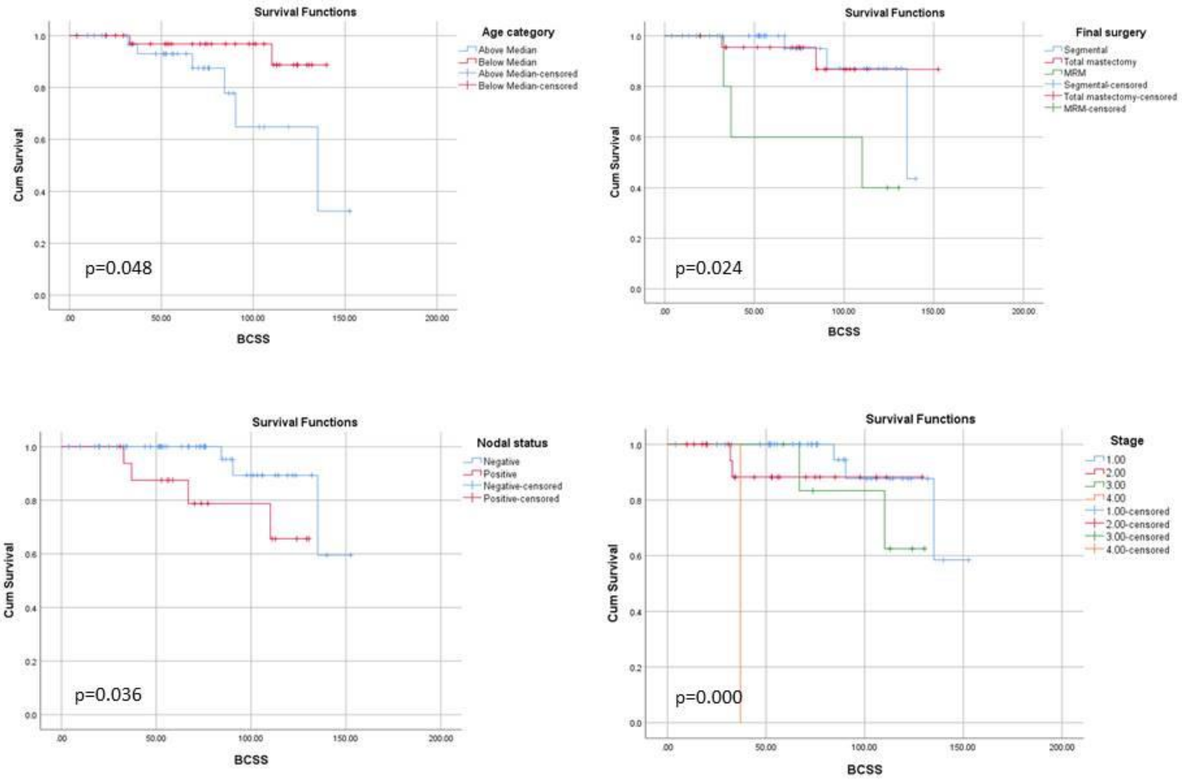


Supplementary figure 2: Variables significantly associated with breast cancer-specific survival (BCSS)

Supplementary table 1: Patient and tumor characteristics of apocrine tumors (n=49)

| | |
|---|---|
| Age in years | |
| Mean | 67 years |
| Median | 66 years |
| Range | 49-98 years |
| Procedure | |
| Segmental | 30 (61%) |
| Total mastectomy | 15 (31%) |
| Modified radical mastectomy | 4 (8%) |
| Tumor size in cm | |
| Mean | 2.0 cm |
| Median | 1.2 cm |
| Range | 0.2 to 10.5 cm |
| Tumor grade | |
| I | 5(10%) |
| II | 39 (80%) |
| III | 5 (10%) |
| Nottingham score | |
| 4 | 1 (2%) |
| 5 | 4 (8%) |
| 6 | 20 (41%) |
| 7 | 19 (39%) |
| 8 | 4 (8%) |
| 9 | 1 (2%) |
| LN status | |
| Negative | 37 (75%) |
| Positive | 11 (23%) |
| Not available | 1 (2%) |
| pT stage | |
| 1 | 34 (69%) |
| 2 | 11 (23%) |
| 3 | 4 (8%) |
| pN stage | |
| 0 | 37 (76%) |
| 1 | 8 (16%) |
| 2 | 2 (4%) |
| 3 | 1 (2%) |
| Unknown | 1 (2%) |
| AJCC Stage* | |
| I | 32 (65%) |
| II | 12 (25%) |
| III | 5 (10%) |
| HER2 immunohistochemistry | |
| Score 0 | 9 (18.5%) |
| Score 1+ | 9 (18.5%) |
| Score 2+/in-situ hybridization negative | 31 (63%) |

| | |
|---|---|
| Ki-67 index | |
| 1 to 10% | 25 (51%) |
| 11 to 20% | 14 (29%) |
| 21 to 30% | 10 (20%) |
| Stromal TILs | |
| 1 to 10% | 33 (67%) |
| 11 to 30% | 12 (25%) |
| 31% or more | 4 (8%) |
| Tumor type | |
| Apocrine carcinoma | 35 (71%) |
| Carcinoma with apocrine features | 14 (29%) |
| Neoadjuvant chemotherapy | |
| No | 39 (80%) |
| Yes | 10 (20%) |
| Response to neoadjuvant chemotherapy (n=10) | |
| pCR | 1 (10%) |
| Residual Cancer Burden 1 | 0 (0%) |
| Residual Cancer Burden 2 | 5 (50%) |
| Residual Cancer Burden 3 | 4 (40%) |
| Radiation | |
| No | 15 (31%) |
| Yes | 34 (69%) |
| Systemic chemotherapy | |
| No | 20 (40%) |
| Yes | 29 (60%) |
| Recurrence | |
| No | 42 (86%) |
| Yes | 7 (14%) |
| Recurrence type | |
| No recurrence | 42 (86%) |
| Loco-regional | 1 (2%) |
| Distant only | 4 (8%) |
| Local+Distant | 2 (4%) |
| Vital status | |
| Alive | 40 (82%) |
| Died of other causes | 3 (6%) |
| Died of breast cancer | 6 (12%) |

*One case with unknown pN stage was considered as node negative for AJCC staging due to negative clinical nodal status.
